# Construction of a novel selection system for endoglucanases exhibiting carbohydrate-binding modules optimized for biomass using yeast cell-surface engineering

**DOI:** 10.1186/2191-0855-2-56

**Published:** 2012-10-23

**Authors:** Akihito Nakanishi, Jungu Bae, Kouichi Kuroda, Mitsuyoshi Ueda

**Affiliations:** 1 Division of Applied Life Sciences, Graduate School of Agriculture, Kyoto University, Sakyo-ku, Kyoto, 606-8502, Japan

**Keywords:** Biorefinery, Carbohydrate-binding module (CBM), Cellulase, Yeast cell-surface engineering

## Abstract

To permit direct cellulose degradation and ethanol fermentation, *Saccharomyces cerevisiae* BY4741 (*Δsed1*) codisplaying 3 cellulases (*Trichoderma reesei* endoglucanase II [EG], *T. reesei* cellobiohydrolase II [CBH], and *Aspergillus aculeatus* β-glucosidase I [BG]) was constructed by yeast cell-surface engineering. The EG used in this study consists of a family 1 carbohydrate-binding module (CBM) and a catalytic module. A comparison with family 1 CBMs revealed conserved amino acid residues and flexible amino acid residues. The flexible amino acid residues were at positions 18, 23, 26, and 27, through which the degrading activity for various cellulose structures in each biomass may have been optimized. To select the optimal combination of CBMs of EGs, a yeast mixture with comprehensively mutated CBM was constructed. The mixture consisted of yeasts codisplaying EG with mutated CBMs, in which 4 flexible residues were comprehensively mutated, CBH, and BG. The yeast mixture was inoculated in selection medium with newspaper as the sole carbon source. The surviving yeast consisted of RTSH yeast (the mutant sequence of CBM: N18R, S23T, S26S, and T27H) and wild-type yeast (CBM was the original) in a ratio of 1:46. The mixture (1 RTSH yeast and 46 wild-type yeasts) had a fermentation activity that was 1.5-fold higher than that of wild-type yeast alone in the early phase of saccharification and fermentation, which indicates that the yeast mixture with comprehensively mutated CBM could be used to select the optimal combination of CBMs suitable for the cellulose of each biomass.

## Introduction

Recently, the production of energy without fossil resources has become necessary to establish a sustainable society because of expanded energy demand, limitations of oil drilling, and environmental pollution (Ge et al. [2011]; [Gerngross and Slater 2000]; Stöcker [2008]). Biorefinery is an important concept because it uses the biomass in a natural cycle (Bouaid et al. [2010]; FitzPatrick et al. [2010]; López et al. [2010]). Bioethanol is a popular energy resource in biorefinery; however, current biorefineries mainly use grain biomass to produce biofuels, which competes with food supply (Ferreira et al. [2010]). Thus, use of biomass garbage has attracted attention for biorefineries. Biomass garbage is an important resource to produce bioethanol because it is mainly composed of cellulose as a carbon source (Chandel and Singh 2011; Wu et al. [2011]). However, most cellulose-degrading techniques require expensive infrastructure, intensive energy, hard chemicals, and the separation of saccharification and fermentation processes. Thus, innovation is necessary to solve these problems.

Yeast cell-surface engineering enables 10^4^–10^5^ enzymes to be displayed on the surface of a yeast cell (Kuroda et al. [2001,2011]; Lin et al. [2003]; Nishitani et al. [2010]; Shibasaki et al. [2001]; Washida et al. [2001]; Ye et al. [2000]). The displaying yeast can be used as a whole-cell biocatalyst without requiring enzyme-separation and -purification processes, and it can immediately take in glucose degraded from cellulose; thus, it is hardly contaminated by other organisms. Furthermore, direct ethanol fermentation from cellulose could be conducted by using cellulase-displaying yeast (Fujita et al. [2002,2004]; Murai et al. [1998]).

Cellulases mainly consist of endoglucanase (EG), cellobiohydrolase (CBH), and β-glucosidase (BG) (Hess et al. [2011]; Todaka et al. [2010]). EG degrades cellulose at endo points, and degradation by EG is important in the early phase of cellulose degradation. The degrading activity of EG is largely supported by the carbohydrate-binding module (CBM), which is matched to the cellulose in each biomass (Ståhlberg et al. [1991]). Furthermore, cellulose degradation is efficiently supported by CBMs because EGs with different CBMs have different binding specificities; CBM is probably required for each biomass in the degrading phase. Thus, it should be possible to improve the cellulase activity when a suitable combination of CBMs is optimized for each cellulose.

In many CBM families, the family 1 CBM is important because it can bind to crystalline cellulose in the early degrading phase, which is important to promote cellulose degradation (Fujita et al. [2004]). Thus far, most studies have focused on 3 conserved amino acid residues to improve the binding ability of the CBM (Fukuda et al. [2006]). In contrast, flexible amino acid residues have rarely been investigated, even though these residues may affect the binding specificity for the cellulose of each biomass.

In this study, the amino acid sequences of family 1 CBMs were compared, and the flexible sequences were determined. To select the optimal combination of CBMs of *Trichoderma reesei* EG IIs for cellulose degradation, a yeast mixture with comprehensively mutated CBM was constructed. The mixture consisted of yeasts codisplaying EG with mutated CBM, in which 4 flexible residues were comprehensively mutated, *T. reesei* CBH II, and *Aspergillus aculeatus* BG I. The yeast mixture was first inoculated into the selection medium with newspaper as a sole carbon source. After selection, the combination of yeasts displaying EG with mutated CBM, CBH, and BG was applied to direct fermentation of newspaper.

## Materials and methods

### Strains and media

*Escherichia coli* strain DH5α (F^–^, 80d*lacZΔ*M15, *Δ*[*lac*ZYA-*arg*F]U169, *end*A1, *hsd*R17[*r*_*k*_^−^, *m*_*k*_^+^], *sup*E44, *thi*-1, *λ*–, *rec*A1, *gyr*A96, *rel*A1, *deo*R) was used as a host for recombinant DNA manipulation and grown in Luria-Bertani medium (1% [*w*/*v*] tryptone, 0.5% [*w/v*] yeast extract, and 1% [*w/v*] sodium chloride) containing 100 μg/mL ampicillin.

*S. cerevisiae* strain BY4741 (*Δsed1*), (*MAT***a**, *his3Δ1*, *leu2Δ0*, *met15Δ0*, *ura3Δ0, YDR077w::KanMX4*), which was obtained from EUROSCARF (Frankfurt, Germany), was used for the cell surface display of cellulase. Yeast transformants were aerobically cultured in synthetic-dextrose (SD) medium (0.67% [*w*/*v*] yeast nitrogen base without amino acids, 2% [*w/v*] glucose, and 0.003% [*w*/*v*] l-methionine) as pre-incubation medium and in SD with casamino acids (SDC) medium (0.67% [*w*/*v*] yeast nitrogen base without amino acids, 2% [*w/v*] glucose, 0.5% [*w/v*] casamino acids, 0.003% [*w*/*v*] l-methionine) as main incubation medium for saccharification and fermentation.

Optimal combination of yeasts displaying EG with mutated CBM was selected from the yeast mixture with comprehensively mutated CBM in a yeast nitrogen base (0.67% [*w*/*v*] yeast nitrogen base without amino acids and 0.003% [*w*/*v*] l-methionine) with 1% newspaper (YNB-newspaper medium).

Yeast fermentation was conducted in 50 mM citric acid buffer (pH 5.0) containing a yeast nitrogen base with casamino acids (0.67% [*w*/*v*] yeast nitrogen base without amino acids, 0.5% [*w/v*] casamino acids and 0.003% [*w*/*v*] l-methionine) with 0.4% [*w*/*v*] laccase-treated newspaper. The medium was termed “yeast nitrogen base with casamino acid (YNBC) medium” .

### Preparation of laccase-treated newspaper

Newspaper was sterilized with 99.5% ethanol (Nakanishi et al. [2012b]), and the volume of the ethanol was 20-fold of the newspaper mass. After sterilization, the newspaper was dried and treated with laccase DAIWA Y120 (4.3 POU/mL) in 50 mM sodium acetate buffer (pH 5.0) for 24 h as pretreatment. The newspaper was then washed with sterilized water and dried for fermentation.

### Plasmid construction

Three plasmids to display EG (pEG), CBH (pCBH), and BG (pBG) were constructed in the previous study (Nakanishi et al. [2012a,b]).

### Determination of the flexible amino-acid residues in family 1 CBMs

The amino acid sequences of 92 family 1 CBMs were compared. All sequence data of family 1 CBMs were obtained from BLAST (http://blast.ncbi.nlm.nih.gov/Blast.cgi). After comparison, the amino acid residues for which the most frequent amino acid appeared with a frequency of less than 40% were selected as the flexible amino-acid residues (Table 1).

**Table 1:** Comparison of CBM amino-acid sequences of EG in family 1 CBMs (92 samples)

**No.**	**A.A.**	**remarks column**
5	W	W(61%), Y(39%)
6	G	G(91%), QA(3%), Y(2%)
7	Q	Unchanging
8	C	Unchanging
9	G	Unchanging
10	G	Unchanging
11	I	I(52%), Q(20%), S(14%), N(7%), T(4%), ARL(1%)
12	G	G(92%), N(9%), S(1%)
13	W	W(74%), Y(23%), F(3%)
14	S	T(52%), S(42%), N(3%), IK(1%)
15	G	Unchanging
16	P	P(74%), A(17%), S(4%), L(3%), Q(2%)
17	T	Unchanging
18	N	T(28%), N(13%), S(12%), AV(11%), QA(5%), RECT(3%), DKI(1%)
19	C	Unchanging
20	A	A(49%), V(36%), Q(5%), ET(4%), D(2%)
21	P	S(66%), A(21%), P(8%), T(4%), G(1%)
22	G	G(86%), P(13%), S(1%)
23	S	S(35%), A(25%), T(17%), Y(12%), FNL(3%), WG(1%)
24	A	T(67%), A(14%), V(9%), C(5%), S(3%), K(2%)
25	C	Uchanging
26	S	T(27%), Q(24%), S(22%), V(10%), H(8%), K(4%), AE(2%), M(1%)
27	T	V(38%), T(20%), K(17%), S(8%), Y(5%), AE(4%), IRQH(1%)
28	L	L(53%), Q(13%), YV(8%), I(6%), S(5%), T(3%), GAWM(1%)
29	N	N(97%), S(2%), G(1%)
30	P	P(56%), D(25%), AS(5%), QE(4%)
31	Y	Y(75%), W(22%), F(2%), A(1%)
32	Y	Y(99%), H(1%)
33	A	S(71%), A(20%), Y(4%), H(3%), F(2%)
34	Q	Uchanging
35	C	Uchanging

Amino-acid numbers (No.) and sequences (A.A.; 1-letter-notation) of the CBM of EG; remarks column, the results of amino-acid comparisons in family 1 CBM; underline: the flexible residue (the conservation < 40%).

### Construction of a yeast mixture codisplaying EG with comprehensively mutated CBM, CBH, and BG

All primers used for plasmid construction are listed in Table 2.

**Table 2:** Primers to construct the comprehensively mutated CBM and to confirm the mutated CBM-sequences

**Primer**	**Sequence**
pEGout F	5’-CGTAGGTCCGCTCCAACCAATACC-3’
pEGout R	5’-CTCAATCCTTATTATGCGCAATGTATTCCG-3’
NNK primer	5’-TTGGAGCGGACCTACGNNKTGTGCTCCTGGCNNKGCTTCTNNKNNKCTCAATCCTTATTATGCGCAATGT-3’
NNK rev	5’-ACATTGCGCATAATAAGGATTGAG-3’
EGct F1	5’-CCAGTGTGGAGGTATTGGTTGGAGCGGACCTACG-3’
EGct R1	5’-GTCGAAGTGGTGATAGTAGTGGCTCCCGGAATACATTGCGCATAATAAGGATTGAG-3’
EGct F2	5’-ATAGATCTCAGCAGACTGTCTGGGGCCAGTGTGGAGGTATTGG-3’
EGct R2	5’-GCACGCGTGGTGGTGGTTGGACCGGATGGTGGCCGGGTCGAAGTGGTGATAGTAGTGGC-3’
CHK	5’-CATGCAACTGTTCAATTTGCCATTG-3’
EGII360bp rev	5’-AGGATAAACCTTCGAGGTAACGCAAGTGCC-3’

To construct the yeast mixture with comprehensively mutated CBM, the flexible amino acid residues (i.e., those at positions 18 of N, 23 of S, 26 of S, and 27 of T) were comprehensively mutated by yeast homologous recombination. First, the 2 double-stranded DNA fragments, i.e., the insert and the vector, were amplified by polymerase chain reaction (PCR). A single-stranded DNA fragments that included NNK sequences (N = A or C or G or T; K = G or T) at the flexible amino acid residues of the CBM of EG were synthesized and amplified as a double-stranded DNA fragment using the NNKrev primer via the Klenow fragment (Toyobo, Osaka, Japan). The DNA fragments were extended with the EGct F1 primer and EGct R1 primer, and further extended with the EGct F2 primer and EGct R2 primer; the product was then used as the insert fragment. The linearized pEG without the flexible amino acid-encoding region in the CBM of EG was amplified with the pEGout F primer and pEGout R primer, and the product was used as the vector fragment. The insert fragments and the vector fragment were simultaneously introduced into yeast codisplaying CBH and BG for homologous recombination. The transformants were named as the yeast mixture with comprehensively mutated CBM.

### Yeast transformation

Plasmid introduction into BY4741 (*Δsed1*) cells was performed using the lithium acetate method (Ito et al. [1983]) with a YEASTMAKER yeast transformation system (Clontech Laboratories, CA, USA). The transformants were isolated on a selective SD medium plate at 30°C for 2–3 days.

### Selection of optimal combination of CBMs

**Figure 1:**
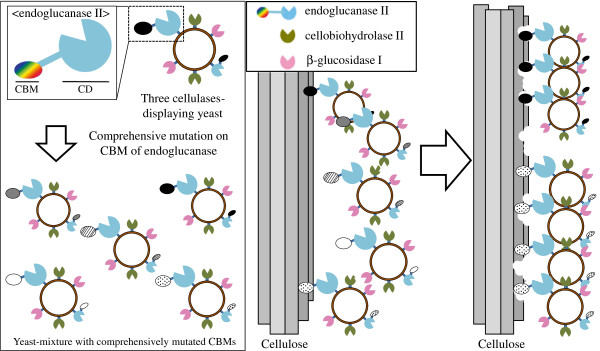
**Illustration of the selection of optimal combination of yeasts displaying EG with mutated CBM from the yeast mixture displaying 3 cellulases including EG with comprehensively mutated CBM.** The yeast mixture consists of yeasts codisplaying 3 cellulases: EG with comprehensively mutated CBM, CBH, and BG. The optimal combination of yeasts displaying EG with mutated CBM for the degradation of the biomass was selected in a medium that contained biomass cellulose as the only carbon source. CBM: Carbohydrate-Binding Module, CD: Catalytic Domain. The optimal combination of yeasts displaying EG with mutated CBM, CBH, and BG could survive because the yeasts could efficiently degrade cellulose and produce glucose.

**Figure 2:**
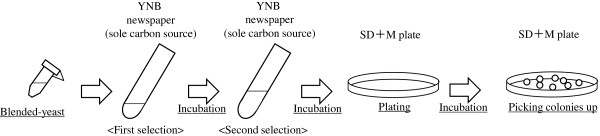
**Scheme to select optimal combination of yeasts displaying EG with mutated CBM.** The selection was conducted twice using the yeast mixture displaying 3 cellulases including EG with comprehensively mutated CBM in a medium containing laccase-treated newspaper as the sole carbon source. After selection, the medium containing the yeast and newspaper was directly transferred to SD+M medium plate. The surviving yeasts were obtained for sequencing.

Optimal combination of yeasts displaying EG with mutated CBMs was selected from the yeast mixture with comprehensively mutated CBMs in a medium that contained biomass cellulose (newspaper etc.) as a sole carbon source (Figure 1). In this study, the yeast mixture with comprehensively mutated CBM was incubated in YNB-newspaper medium for 1 week twice in a row. The yeast and the newspaper were then transferred to SD+M agarose plates to isolate the surviving yeasts (Figure 2). The CBM-encoding DNAs of the surviving yeasts were amplified by colony-direct PCR using the CHK primer and EGII360bp rev primer (Table 2) and sequenced after purification by using the ethanol-precipitation method.

### Immunofluorescence labeling of yeast cells and observation

Immunofluorescence labeling of cells was performed according to the previously described method (Kobori et al. [1992]). Three monoclonal antibodies were used as the primary antibodies at a dilution of 1:300: mouse monoclonal anti-FLAG M2 antibody (Sigma-Aldrich, MO, USA) for EG, mouse monoclonal StrepMAB-Classic antibody (IBA, MO, USA) for CBH, and mouse monoclonal anti-RGS-His antibody (QIAGEN, Hilden, Germany) for BG. The mixtures of cells and the respective antibodies were incubated at room temperature with gentle shaking for 1.5 h, and the cells were washed with phosphate-buffered saline (PBS; pH 7.4). As the secondary antibody, Alexa Fluor 488 anti-mouse IgG (Invitrogen, CA, USA) was used at a dilution of 1:300 at room temperature with gentle shaking for 1.5 h. The cells were washed with PBS (pH 7.4) and observed with an inverted microscope IX71 (Olympus, Tokyo, Japan) through a U-MNIBA2 mirror unit with a BP470–490 excitation filter, a DM505 dichroic mirror, and a BA510-550 emission filter (Olympus). Live images were obtained using Aqua Cosmos 2.0 software (Hamamatsu Photonics, Shizuoka, Japan) to control a digital change-coupled device camera (Hamamatsu Photonics).

### Measurement of yeast fermentation activity

After pre-cultivation in SD medium for 48 h at 30°C, each yeast cell was aerobically cultivated for 60 h at 30°C in SDC medium. The cells were collected by centrifugation for 5 min at 5000 × *g* and 4°C, and washed with PBS (pH 7.4). To confirm the result of the selection for the newspaper from the yeast mixture with comprehensively mutated CBM, RTSH yeast (4 amino acid residues of the CBM were mutated: N18R, S23T, S26S, and T27H) and wild-type yeast (the CBM was not mutated) were mixed in a ratio of 1:46 and a total OD_600_ of 10 in YNBC medium. The yeasts were termed “blended yeasts”. CO_2_ gas was injected into the reaction vessel for 2 min to displace O_2_. For fermentation, the blended yeasts were semi-aerobically cultured in YNBC medium stirred by a magnetic bar rotating at 130 rpm at 30°C. Five hundred microliters of reacting medium was collected and filtered using Ultrafree-MC Centrifugal Filter Units (Millipore, MA, USA) for ethanol quantification. The produced ethanol was quantified by using a high-performance liquid chromatography (HPLC) system that consisted of a LC-20 AD pump (Shimadzu, Kyoto, Japan), a CTO-20A column oven (Shimadzu), a RID-10A detector (Shimadzu), a YMC-Pack Polyamine II column (4.6 × 250 mm) (YMC Co. Ltd., Kyoto, Japan), and a 7725 injector (Rheodyne, CA, USA). The concentration of produced ethanol was determined from the chromatographic data monitored by the RID-10A, and the results were processed using LC Solution software (Shimadzu). The mobile phase was water and acetonitrile mixed in a ratio of 5:95 as isocratic, and the temperature of the column oven was set at 30°C. The flow rate was 1.0 mL/min.

## Results

### Comparison of the sequences of family 1 CBMs

Conserved and flexible amino acid residues were determined by comparing the amino acid sequences of 92 types of family 1 CBMs (Table 1). More than 99% of the 5th, 31st, and 32nd amino acids of the CBMs were aromatic amino acids that bind to the flat surface of crystalline cellulose; the 7th, 8th, 9th, 10th, 15th, 17th, 19th, 25th, 34th, and 35th amino acids were conserved as skeletal elements, where the 19th and 35th amino acids as well as the 8th and 25th amino acids formed disulfide bonds with each other without any exception. The frequency of appearance of the major amino acids at the 18th, 23rd, 26th, and 27th positions was lower than 40%, and we decided that they were flexible amino acid residues.

### Construction of a yeast mixture with comprehensively mutated CBM

To construct the yeast mixture with comprehensively mutated CBM, the flexible amino acid residues were comprehensively mutated. The DNA sequences focused on the 18th, 23rd, 26th, and 27th amino acids were evaluated to confirm the construction (Table 3). We concluded that the sequence of the CBM region in EG was comprehensively mutated because the null hypothesis for the appearance of each amino acid was rejected (appearance value < 0.05).

**Table 3:** Confirmation of appearance of amino acids in comprehensively mutated CBM in 88 samples

**A.A. number; 18**		**A.A. number; 23**		**A.A. number; 26**		**A.A. number; 27**	
A.A.	Rate	A.A.	Rate	A.A.	Rate	A.A.	Rate
T	10 (11.4%)	S	12 (13.6%)	L	10 (11.4%)	L	11 (12.5%)
S	9 (10.2%)	G	9 (10.2%)	V	8 (9.1%)	R	9 (10.2%)
A	9	L	7 (8.0%)	S	8	S	8 (9.1%)
L	7 (8.0%)	T	7	R	7 (8.0%)	T	7 (8.0%)
P	7	R	7	W	6 (6.8%)	G	6 (6.8%)
F	6 (6.8%)	A	6 (6.8%)	I	5 (5.7%)	C	5 (5.7%)
R	6	M	6	Stop	5	P	5
E	5 (5.7%)	W	5 (5.75)	G	5	W	4 (4.5%)
Y	4 (4.55)	H	4 (4.5%)	T	4 (4.5%)	V	4
V	3 (3.4)	Q	4	P	4	A	3 (3.4%)
Stop	3	N	3 (3.4%)	Y	4	F	3
N	3	D	3	A	3 (3.4%)	D	3
K	3	I	3	M	3	M	3
C	2 (2.3%)	F	2 (2.3%)	F	3	H	3
W	2	K	2	C	3	K	3
G	2	V	2	N	2 (23%)	Stop	3
Q	2	P	2	E	2	I	2 (2.3%)
I	2	Y	2	Q	2	Q	2
D	2	E	1 (1.1%)	K	1 (1.1%)	Y	2
M	1 (1.1%)	Stop	1	H	1	N	1 (1.1%)
				F	1	E	1
				D	1		

### Screening of optimal combination of CBMs

We performed screening to select the optimal combination of yeasts displaying EG with mutated CBM from the yeast mixture with comprehensively mutated CBM using newspaper as the sole carbon source. After the incubation of the yeast mixture in the medium containing newspaper, yeasts were spread on a SD+M plate. Colonies were formed by the surviving yeast after 2–3 days, and the CBM-encoding DNAs in the surviving yeasts on the plate were sequenced. The surviving yeasts consisted of RTSH yeast (the sequence of the CBM of EG among displayed 3 cellulases was mutated: N18R, S23T, S26S, and T27H) and wild-type yeasts (the sequence of the CBM of EG was not changed) in a ratio of 1:46. These results suggest that the combination of RTSH yeast and wild-type yeast could be optimal for the degradation of cellulose in newspaper.

### Confirmation of the display of EG with mutated CBM, CBH, and BG

**Figure 3:**
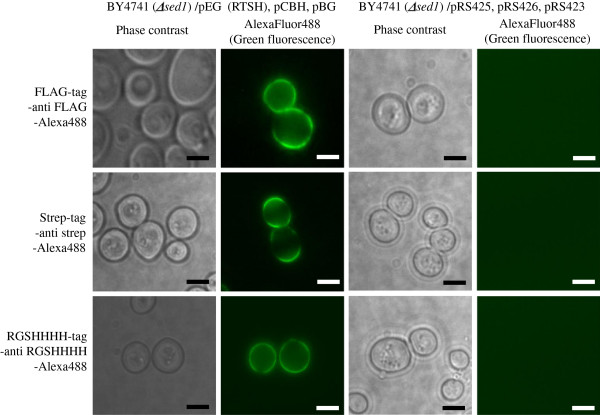
**Fluorescence observation of RTSH yeast, harboring EG with CBM having RTSH mutation, CBH, and BG, after immunofluorescence labeling.** Yeast cells were labeled with the following antibodies: mouse monoclonal anti-FLAG M2 antibody for EG (upper column), mouse monoclonal StrepMAB-Classic antibody for CBH (middle column), mouse monoclonal anti-RGS-His antibody for BG (lower column), and Alexa Fluor 488 anti-mouse immunoglobulin. The left column represents RTSH yeast and the right column represents yeast harboring pRS423, pRS425, and pRS426 (control vectors). Phase-contrast micrographs are presented in the left column and fluorescence micrographs are presented in the right column. The scale bar is 5 μm.

Immunofluorescence labeling was performed with the 3 different antibodies to confirm the display of cellulases including EG with mutated CBM on the yeast cell surface. The green fluorescence of Alexa Fluor 488 for each tag was observed on the cell surface of each transformant (Figure 3), and each cellulase was co-displayed on the yeast cell surface (data not shown). This result indicated that the 3 cellulases (EG with mutated CBM, CBH, and BG) were successfully displayed on the surface of RTSH yeast.

### Fermentation activity of the mixture of RTSH yeast and wild-type yeast

**Figure 4:**
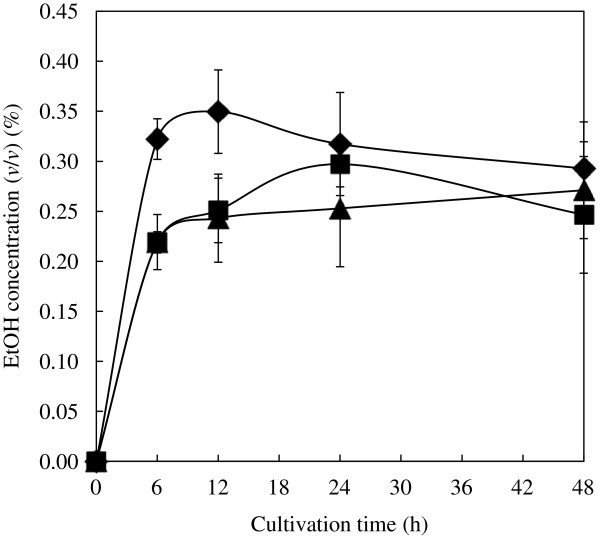
**Comparison of ethanol fermentation by the blended yeast of RTSH and wild-type yeast with that by RTSH yeast alone and wild-type yeast alone.** Ethanol fermentation was evaluated using high-performance liquid chromatography as described in the Materials and methods section. The carbon source for fermentation was laccase-treated newspaper. The symbols indicate each strain: ♦, the blended yeast of wild-type yeast and RTSH yeast in a ratio of 46:1;  , RTSH yeast; ▲, wild–type yeast. Values represent the means ± standard deviation of the results from 3 independent experiments.

To confirm the result of screening that the combination of RTSH and wild-type yeast in a ratio of 1:46 is optimal for degradation of newspaper, we performed direct fermentation of newspaper. The fermentation activities of the blended yeast (the combination of RTSH yeast and wild-type yeast), RTSH yeast alone, and wild-type yeast alone were compared (Figure 4). The activity of the blended yeast was 1.48-fold higher than that of wild-type yeast at 6 h. The activity of RTSH yeast was almost equal to that of wild-type yeast at 6 h; however, the activity of RTSH yeast was 1.21-fold higher than that of wild-type yeast at 24 h. These results indicate that the saccharification activity of blended yeast which we obtained from screening was higher than that of wild-type yeast alone and the screening system is suitable for selection of optimal combination of CBMs.

## Discussion

In this study, 92 amino acid sequences of family 1 CBMs were compared, and conserved and flexible sequences were determined (Table 1). The conserved sequences contained components of the peptide skeletal structure such as disulfide bonds between amino acids 19 and 35 as well as 8 and 25 in addition to the planar binding face (mostly aromatic amino acids, i.e., residues 5, 31, and 32). Furthermore, the distance between the aromatic amino acids was highly conserved (approximately 10.4 Å) (Linder et al. [1995]). Most of the previous reports have focused on these conserved region to enhance the binding ability of CBM (Fukuda et al. [2006]). In contrast, the variability of amino acids at positions 18, 23, 26, and 27 indicates that these residues are not necessary for the main structure of the CBM. Rather, they may have been tuned to obtain an optimal structure to bind to each biomass cellulose for efficient degradation in many microorganisms. Thus, in this study, we focused on the flexible region to obtain optimal combination of CBM for various types of biomass. A yeast mixture with CBM that were comprehensively mutated at the flexible residues was constructed to select the optimal combination of CBMs for each biomass cellulose, and the comprehensive mutation was confirmed by the rejection of the null hypothesis for each amino acid (Table 3).

The yeast mixture was cultivated and screened in YNB-newspaper medium to obtain the optimal combination of yeasts displaying EG with mutated CBM. The survived yeasts consisted of RTSH yeast and wild-type yeast in a ratio of 1:46, and the blended yeast (the combination of RTSH yeast and wild-type yeast) produced 1.48-fold more ethanol than wild-type yeast alone at 6 h. The amount of ethanol produced by RTSH yeast alone was almost equal to that produced by wild-type yeast alone at 6 h. Interestingly, RTSH yeast produced 1.21-fold more ethanol than wild-type yeast at 24 h (Figure 4). The higher fermentation rate of RTSH yeast indicates that the saccharification of cellulose by RTSH yeast might be faster than that by wild-type yeast. Thus, CBM with RTSH mutation is probably more efficient than wild-type CBM for partial degradation. Attractively, only 1 RTSH yeast was obtained during the selection process, in contrast to 46 wild-type yeasts, which indicates that RTSH yeast could have helped wild-type yeast to degrade the cellulose of newspaper in the early phase of cellulose degradation. Then, the screening was also performed on hydrothermally processed rice straw, and RTSH yeast (N18R, S23T, S26S, and T27H), RPQA yeast (N18R, S23P, S26Q, and T27A), wild-type yeast, and RPLA yeast (N18R, S23P, S26L, and T27A) were obtained in a ratio of 10:7:2:1 (data not shown). The difference in composition depending on the substrate could be due to variation of the cellulose structure in different types of biomass, indicating the feasibility of using this screening system to obtain the optimal combination of CBMs.

The present study is the first to identify flexible residues in family 1 CBMs, to construct a selection system for the optimal combination of CBMs of EGs for each type of biomass using a yeast mixture with comprehensively mutated CBM, and to demonstrate improved cellulose saccharification and ethanol fermentation by the selected displaying yeast. Our technique *in vitro* may be used to easily select the optimal combinations of EGs, like cellulosomes *in vivo* (Han et al. [2003]; Nataf et al. [2010]), for various types of biomass.

## Competing interests

The authors declare that they have no competing interests.

## Authors’ contributions

MU, KK, and AN participated in the design of this study. MU and KK supervised this study and edited manuscript. AN and JB performed the experimental work, analyzed the data, and drafted the manuscript. All authors read and approved the final manuscript.
